# Supplementary material for: Transcriptome Profiling of Circulating Tumor Cells to Predict Clinical Outcomes in Metastatic Castration-Resistant Prostate Cancer
Source: Int J Mol Sci. 2023 May 19;24(10):9002. doi: 10.3390/ijms24109002 (PMC10218842; doi:10.3390/ijms24109002)
Supplement: Supplementary file 1 [file ijms-24-09002-s001.zip › Supplementary Figures IJMS_20230518.pdf]

Supplementary Figures

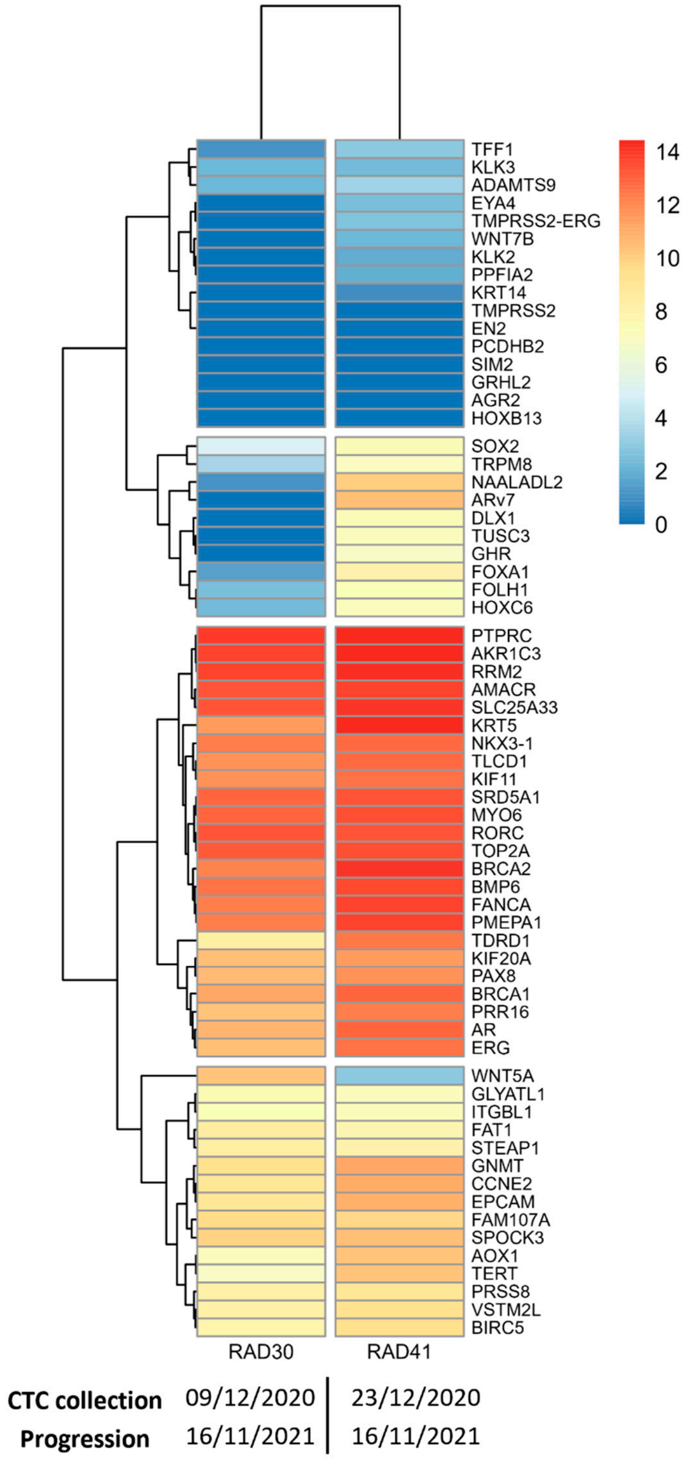

Figure S1) CTC profiles of samples 'RAD30' and 'RAD41' based on the complete gene panel. Samples were collected from a patient receiving PARPi, 'RAD30' at baseline followed by 'RAD41' 14 days later. Expression annotation: log2 expression values.

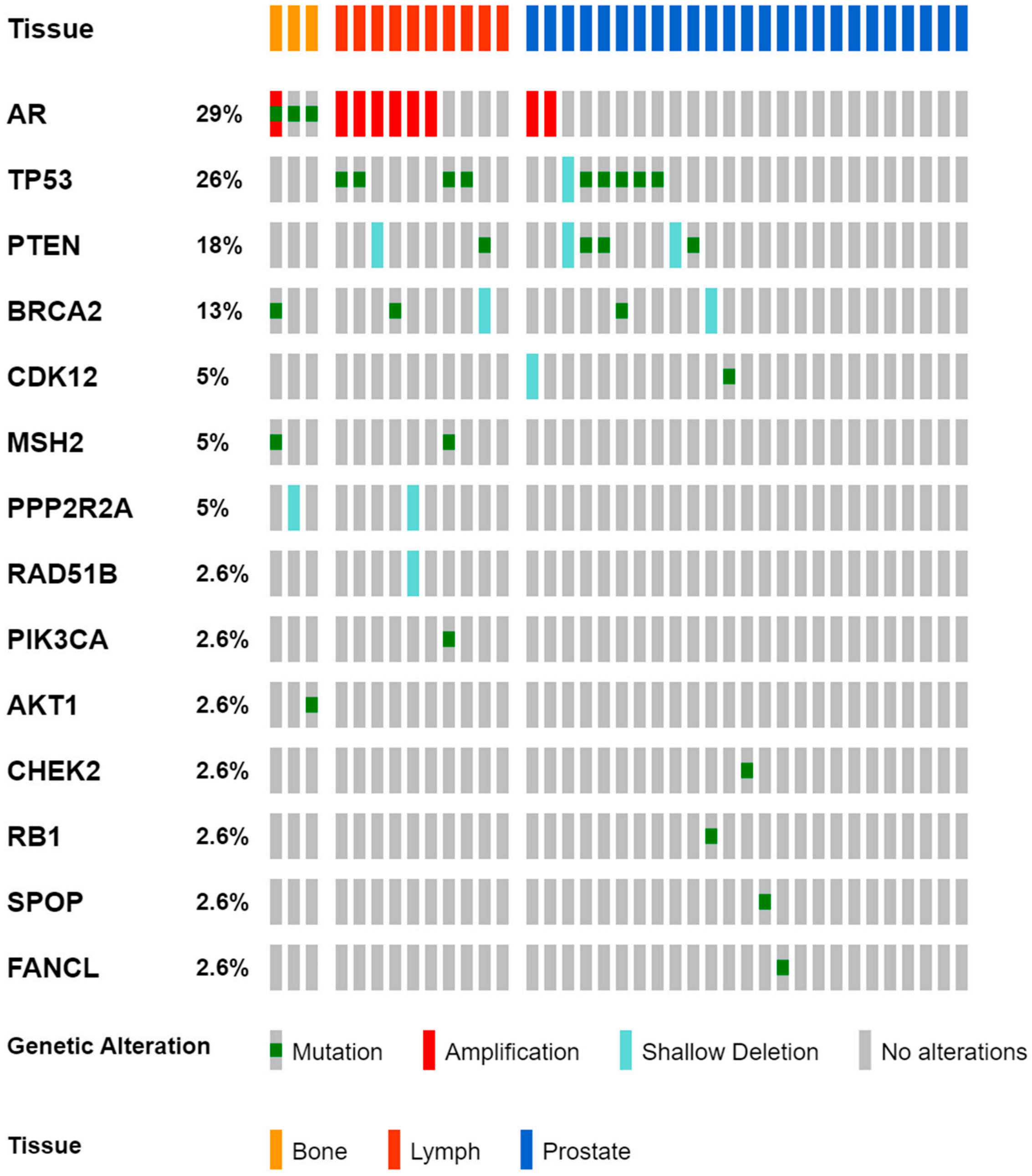

Figure S2) Genetic alterations identified through next generation DNA sequencing in the PROMPT trial. Genetic alterations: Mutation, inframe, missense, splice, or truncal mutations; Amplification, copy number amplification; Shallow Deletion, heterogeneous deletion; No alterations, wildtype. Tissue; Bone, metastatic bone biopsy; Lymph, metastatic lymph-node biopsy; Prostate, prostate tissue biopsy.

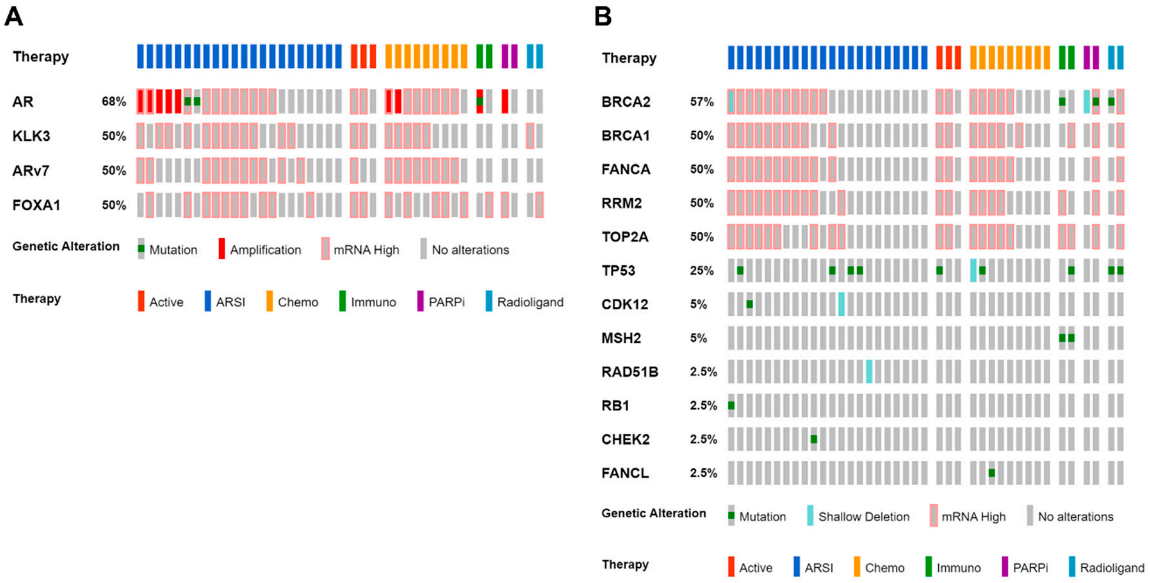

Figure S3) Genetic alterations in tissue biopsies and high gene expression in matched CTC samples of in AR signaling genes (A) and DDR genes (B). Genetic alterations: Mutation, inframe, missense, splice, or truncal mutations in tissue biopsies; Amplification, copy number amplification in tissue biopsies; Shallow Deletion, heterogeneous deletion in tissue biopsies; mRNA High; high expression in CTC samples; No alterations, wildtype gene in tissue biopsies. Therapy; Active, active surveillance; ARSI, ARSI therapy; Chemo, chemotherapy; Immuno, immunotherapy; PARPi, PARPi therapy; Radioligand, radioligand therapy.

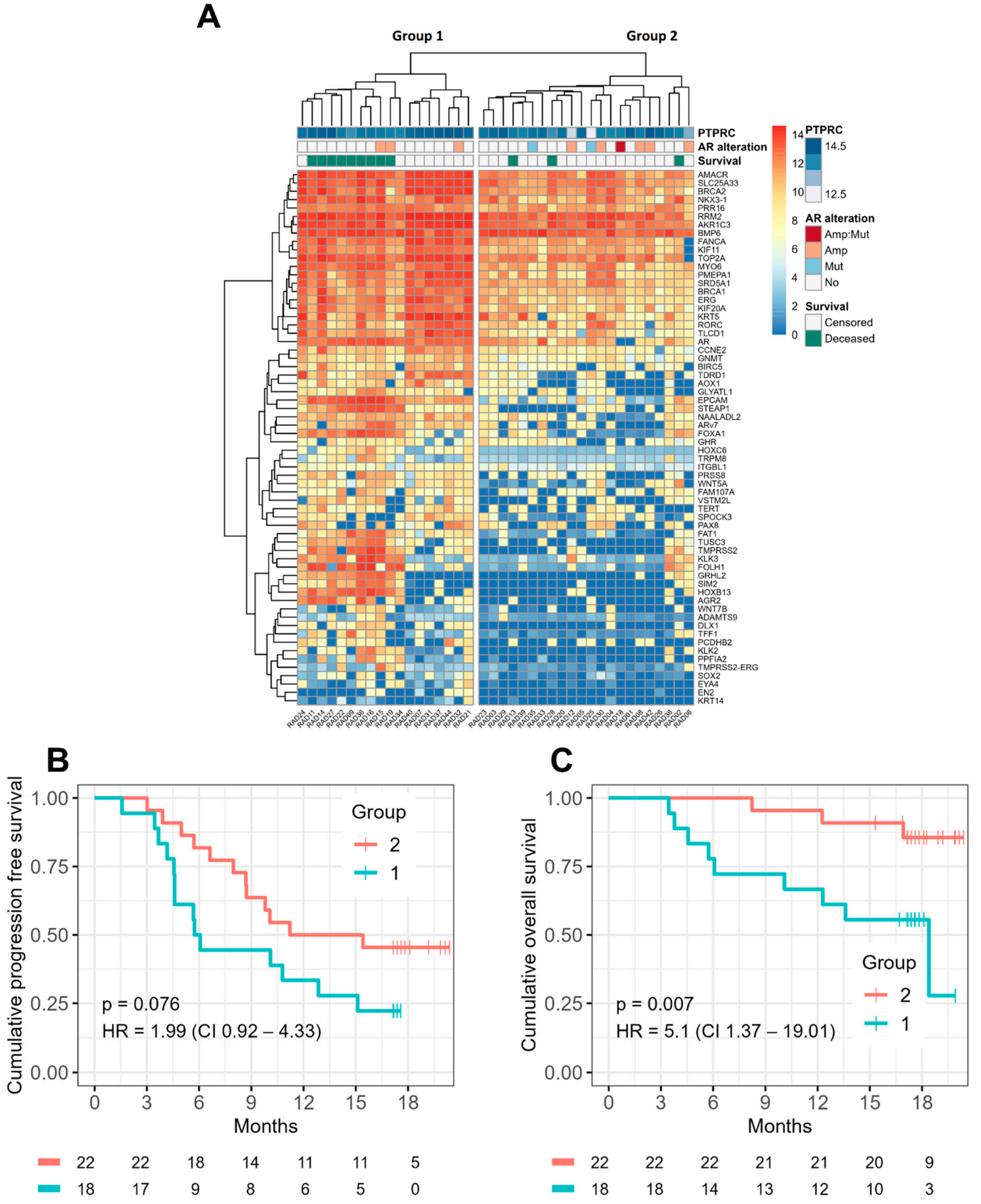

Figure S4) Hierarchical clustering of the therapy-agnostic cohort using the complete gene panel (A). Cluster *p*-values are *p*=0.26 for 'Group 1' and *p*=0.27 for 'Group 2'. PFS (B) and OS (C) of patients with either 'Group 1' and 'Group 2' profiles. Expression annotation: log2 expression values. Heatmap annotation: PTPRC, leukocyte background signal in each sample; AR alteration, Amp:Mut, AR amplification and mutation, Amp, AR amplification, Mut, AR mutation, No, no alteration; Survival, Censored, alive at last follow-up, Deceased, deceased at last follow-up. Survival statistics: HR, Hazard ratio; CI, 95% confidence interval; *p*, *p*-value.

Endpoint	Genes	HR	CI	p
PFS	SPOCK3:High	0.83	(0.38–1.79)	0.626
	KRT5:High	0.90	(0.42–1.94)	0.782
	EN2:High	0.97	(0.37–2.58)	0.954
	RRM2:High	1.06	(0.49–2.30)	0.877
	BMP6:High	1.09	(0.50–2.36)	0.825
	TMPRSS2-ERG:High	1.10	(0.51–2.37)	0.813
	BRCA1:High	1.16	(0.54–2.52)	0.702
	BRCA2:High	1.18	(0.54–2.55)	0.681
	AKR1C3:High	1.18	(0.54–2.55)	0.681
	TERT:High	1.19	(0.55–2.57)	0.657
	PMEPA1:High	1.20	(0.55–2.59)	0.650
	ARv7:High	1.26	(0.58–2.74)	0.551
	TDRD1:High	1.30	(0.60–2.82)	0.502
	FAM107A:High	1.30	(0.60–2.84)	0.506
	KIF20A:High	1.31	(0.61–2.85)	0.487
	CCNE2:High	1.32	(0.61–2.85)	0.483
	PRR16:High	1.33	(0.61–2.87)	0.471
	AOX1:High	1.33	(0.61–2.88)	0.469
	FAT1:High	1.36	(0.63–2.94)	0.435
	ADAMTS9:High	1.42	(0.66–3.08)	0.374
	ERG:High	1.42	(0.66–3.08)	0.374
	GHR:High	1.46	(0.67–3.15)	0.342
	EYA4:High	1.46	(0.68–3.17)	0.334
	AR:High	1.47	(0.68–3.19)	0.324
	PAX8:High	1.50	(0.68–3.27)	0.313
	TOP2A:High	1.52	(0.69–3.31)	0.297
	FANCA:High	1.54	(0.70–3.36)	0.281
	GLYATL1:High	1.54	(0.71–3.35)	0.274
	GNMT:High	1.55	(0.71–3.38)	0.273
	HOXC6:High	1.61	(0.74–3.48)	0.229
	RORC:High	1.62	(0.74–3.53)	0.228
	FOLH1:High	1.73	(0.80–3.76)	0.165
	ITGBL1:High	1.74	(0.80–3.80)	0.164
	KIF11:High	1.80	(0.82–3.94)	0.140
	FOXA1:High	1.85	(0.85–4.03)	0.123
	SOX2:High	1.85	(0.84–4.09)	0.129
	KLK2:High	1.89	(0.86–4.18)	0.116
	WNT7B:High	1.91	(0.87–4.17)	0.105
	PPFIA2:High	1.92	(0.88–4.20)	0.101
	EPCAM:High	1.92	(0.88–4.21)	0.101
	TUSC3:High	1.95	(0.89–4.27)	0.094
	NAALADL2:High	1.99	(0.91–4.36)	0.085
	SRD5A1:High	2.00	(0.90–4.44)	0.087
	AMACR:High	2.03	(0.91–4.50)	0.082
	STEAP1:High	2.07	(0.94–4.53)	0.070
	SLC25A33:High	2.09	(0.94–4.62)	0.070
	VSTM2L:High	2.09	(0.95–4.61)	0.069
	WNT5A:High	2.12	(0.96–4.71)	0.064
	TLCD1:High	2.18	(0.99–4.83)	0.054
	NKX3-1:High	2.22	(1.01–4.86)	0.047
	PCDHB2:High	2.27	(1.02–5.04)	0.044
	SIM2:High	2.32	(1.06–5.07)	0.036
	TFF1:High	2.32	(1.05–5.13)	0.038
	BIRC5:High	2.44	(1.10–5.39)	0.028
	KRT14:High	2.52	(1.16–5.50)	0.020
	TMPRSS2:High	2.68	(1.20–5.97)	0.016
	MYO6:High	2.75	(1.21–6.23)	0.015
	DLX1:High	2.94	(1.33–6.50)	0.008
	PRSS8:High	3.14	(1.35–7.33)	0.008
	HOXB13:High	3.18	(1.45–6.97)	0.004
	TRPM8:High	3.32	(1.45–7.59)	0.004
	AGR2:High	3.48	(1.55–7.84)	0.003
	GRHL2:High	3.99	(1.79–8.93)	<0.001
	KLK3:High	4.01	(1.75–9.19)	0.001

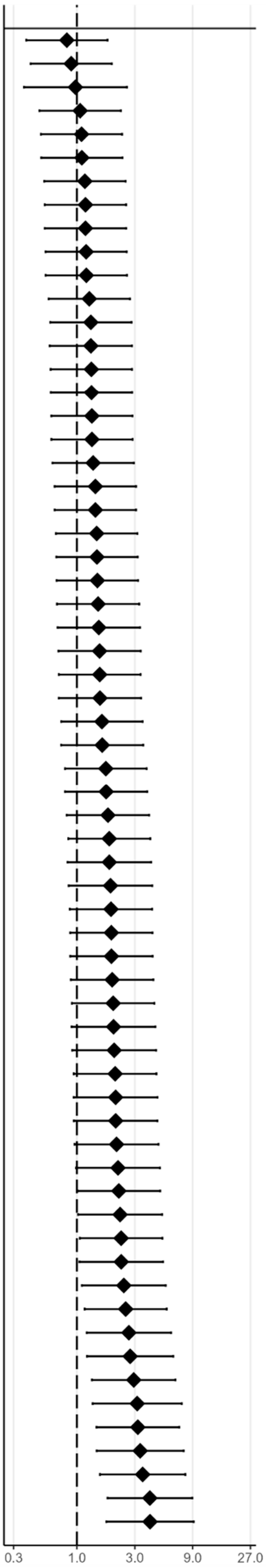

Figure S5) The prognostic value of genes from the complete gene panel on PFS in the therapy-agnostic cohort. A multivariate model was used to determine risk for progression (HR), 95% confidence intervals, and statistical significance (p). Survival statistics: HR, Hazard ratio; CI, Confidence interval; *p*, *p*-value.

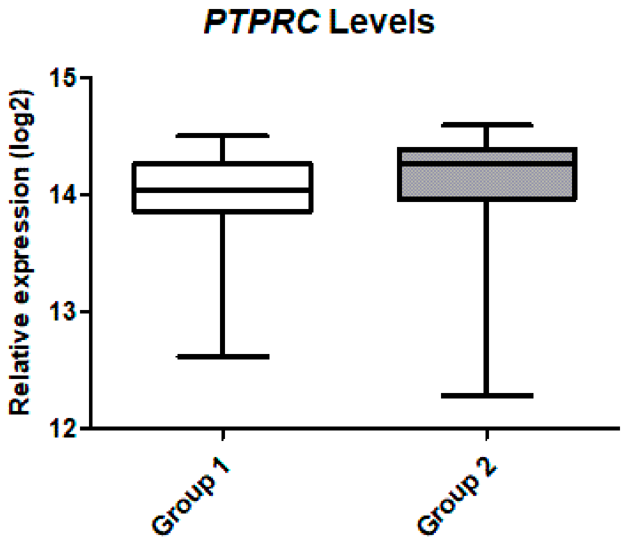

Figure S6) *PTPRC* levels in 'Group 1' (n=16) and 'Group 2' (n=24) patients from therapy-agnostic cohort. Patients were grouped via unsupervised hierarchical clustering using the agnostic gene panel (Figure 2A).

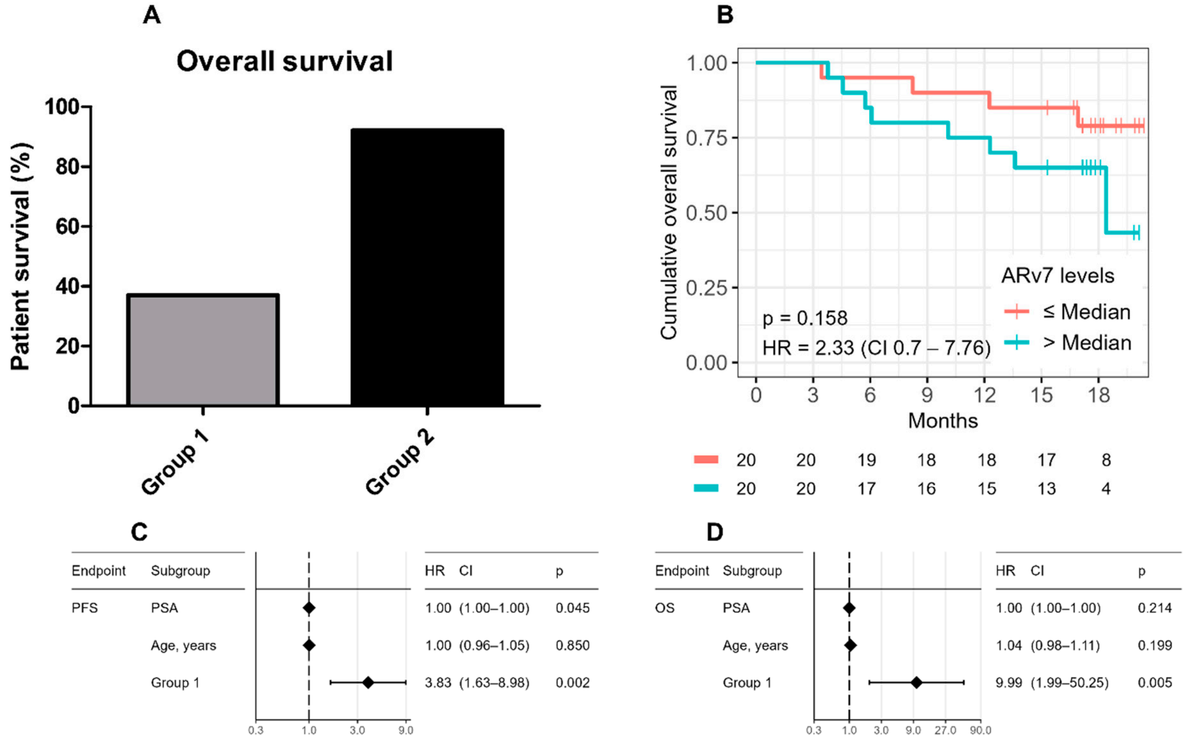

Figure S7) Survival of 'Group 1' (n=16) and 'Group 2' (n=24) patients in the therapy-agnostic cohort at last follow-up (A). The prognostic value of *ARv7* levels on OS in the therapy-agnostic cohort (B). Multivariate analysis on PFS (C) and OS (D) including variables: PSA, age, and the 'Group 1' profile in the therapy-agnostic cohort. Survival statistics: HR, Hazard ratio; CI, 95% Confidence interval; *p*, *p*-value.

Endpoint	Genes	HR	CI	p
PFS	BRCA2:High	0.42	(0.15–1.20)	0.106
	RRM2:High	0.42	(0.15–1.20)	0.106
	BRCA1:High	0.45	(0.16–1.27)	0.131
	SPOCK3:High	0.46	(0.16–1.29)	0.140
	KIF20A:High	0.52	(0.18–1.47)	0.215
	TERT:High	0.58	(0.20–1.62)	0.297
	ADAMTS9:High	0.61	(0.22–1.71)	0.347
	AKR1C3:High	0.61	(0.22–1.70)	0.349
	FANCA:High	0.70	(0.25–1.93)	0.487
	PMEPA1:High	0.70	(0.25–1.93)	0.487
	CCNE2:High	0.70	(0.25–1.93)	0.487
	RORC:High	0.74	(0.27–2.04)	0.561
	KRT5:High	0.74	(0.27–2.05)	0.565
	AOX1:High	0.74	(0.27–2.06)	0.569
	GNMT:High	0.78	(0.28–2.16)	0.633
	WNT7B:High	0.86	(0.31–2.38)	0.770
	ERG:High	0.87	(0.32–2.42)	0.796
	ITGBL1:High	0.90	(0.32–2.49)	0.837
	EN2:High	0.91	(0.25–3.23)	0.878
	BIRC5:High	0.92	(0.33–2.56)	0.879
	HOXC6:High	0.94	(0.34–2.59)	0.897
	PAX8:High	1.15	(0.42–3.20)	0.782
	KIF11:High	1.18	(0.43–3.27)	0.751
	MYO6:High	1.18	(0.43–3.27)	0.751
	PCDHB2:High	1.18	(0.43–3.27)	0.751
	TDRD1:High	1.19	(0.43–3.29)	0.743
	SLC25A33:High	1.20	(0.43–3.31)	0.732
	SRD5A1:High	1.20	(0.43–3.31)	0.732
	TOP2A:High	1.21	(0.44–3.34)	0.718
	TLCD1:High	1.28	(0.46–3.56)	0.631
	AMACR:High	1.28	(0.46–3.56)	0.631
	AR:High	1.28	(0.46–3.56)	0.631
	NKX3-1:High	1.32	(0.48–3.65)	0.594
	BMP6:High	1.32	(0.48–3.66)	0.594
	SOX2:High	1.34	(0.48–3.70)	0.574
	FAT1:High	1.34	(0.48–3.72)	0.572
	WNT5A:High	1.37	(0.49–3.80)	0.547
	KRT14:High	1.38	(0.49–3.88)	0.546
	EYA4:High	1.38	(0.50–3.81)	0.539
	FAM107A:High	1.45	(0.51–4.10)	0.482
	TMPRSS2-ERG:High	1.51	(0.54–4.18)	0.433
	PRSS8:High	1.53	(0.54–4.32)	0.422
	TFF1:High	1.73	(0.62–4.89)	0.298
	DLX1:High	1.74	(0.63–4.86)	0.287
	STEAP1:High	1.76	(0.63–4.90)	0.282
	ARv7:High	1.76	(0.63–4.90)	0.282
	PRR16:High	1.90	(0.67–5.39)	0.226
	NAALADL2:High	2.05	(0.72–5.84)	0.180
	PPFIA2:High	2.13	(0.75–6.06)	0.156
	TMPRSS2:High	2.15	(0.76–6.13)	0.151
	EPCAM:High	2.31	(0.81–6.56)	0.117
	GHR:High	2.37	(0.84–6.73)	0.104
	FOLH1:High	2.40	(0.86–6.74)	0.096
	TUSC3:High	2.44	(0.86–6.96)	0.094
	KLK2:High	2.57	(0.86–7.63)	0.090
	GLYATL1:High	2.72	(0.95–7.82)	0.063
	SIM2:High	2.93	(1.00–8.63)	0.051
	VSTM2L:High	3.00	(1.01–8.90)	0.048
	TRPM8:High	3.33	(1.11–10.03)	0.033
	AGR2:High	3.44	(1.13–10.52)	0.030
	FOXA1:High	3.72	(1.24–11.12)	0.019
	HOXB13:High	4.19	(1.42–12.32)	0.009
	KLK3:High	4.46	(1.45–13.77)	0.009
	GRHL2:High	6.56	(1.93–22.33)	0.003

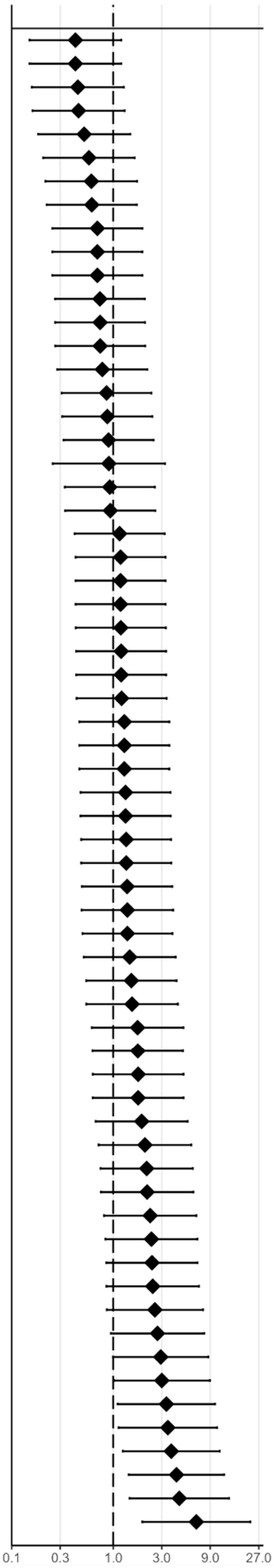

Figure S8) The prognostic value of genes from the complete gene panel on PFS in the ARSI cohort. A multivariate model was used to determine risk for progression (HR), 95% confidence intervals, and statistical significance (p). Survival statistics: HR, Hazard ratio; CI, Confidence interval; *p*, *p*-value.

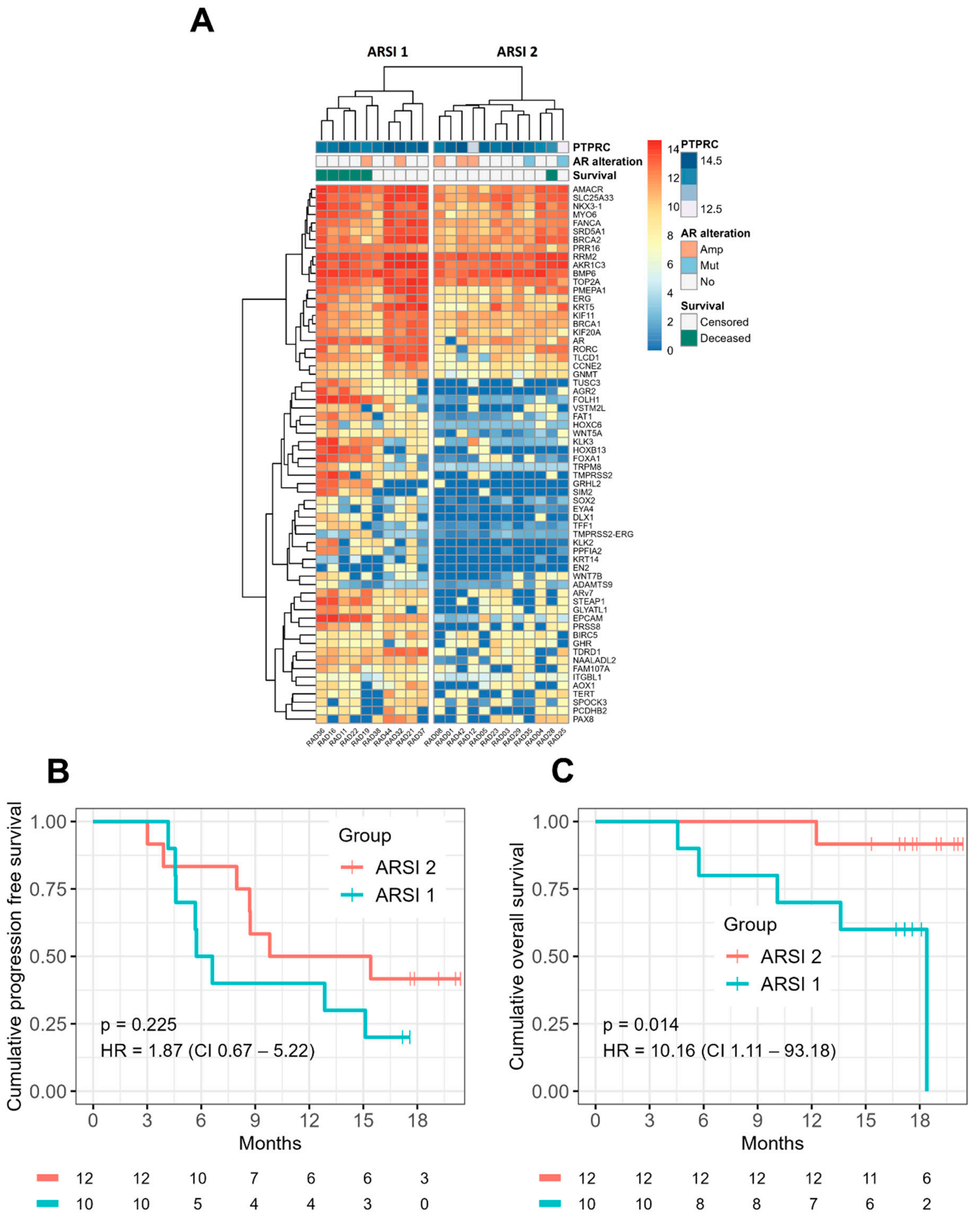

Figure S9) Hierarchical clustering of the ARSI cohort using the complete gene panel (A). Cluster *p*-values are *p*=0.26 for 'ARSI 1' and *p*=0.3 for 'ARSI 2'. Kaplan-Meier plots showing PFS (B) and OS (C) of patients with 'ARSI 1' and 'ARSI 2' profiles. Expression annotation: expression is shown in log2 values, from 0 (dark blue) to 14 (deep red). Heatmap annotations: PTPRC, leukocyte background signal in each sample; AR alteration, Amp, AR amplification, Mut, AR mutation, No, no alteration; Survival, Censored, alive at last follow-up (white), Deceased, deceased at last follow-up survey (green). Survival statistics: HR, Hazard ratio; CI, 95% confidence interval; *p*, *p*-value.

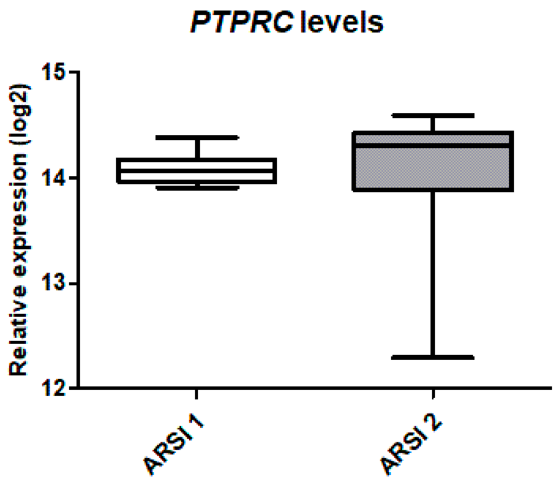

Figure S10) *PTPRC* levels in 'ARSI 1' and 'ARSI 2' patients. Patients were grouped via unsupervised hierarchical clustering using the ARSI gene panel (Fig. 3A).

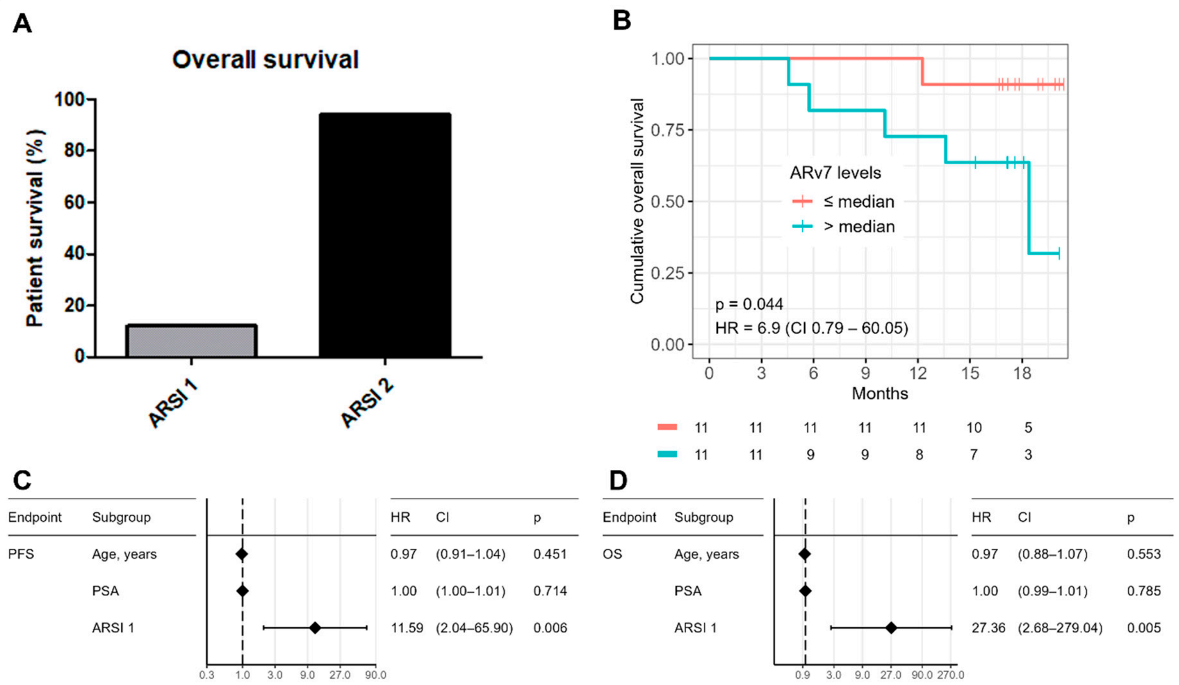

Figure S11) Survival of 'ARSI 1' and 'ARSI 2' patients in the ARSI cohort at last follow-up (A). The prognostic value of *ARv7* levels on OS in the ARSI cohort (B). Multivariate analysis on PFS (C) and OS (D) including variables: PSA, age, and the 'ARSI 1' profile in the ARSI cohort. Survival statistics; HR, Hazard ratio; CI, 95% confidence interval; *p*, *p*-value.

Endpoint	Genes	HR	CI	p
PFS	TMPRSS2-ERG:High	0.25	(0.03–2.27)	0.219
	BMP6:High	0.27	(0.03–2.50)	0.252
	SPOCK3:High	0.60	(0.10–3.65)	0.583
	KRT5:High	0.60	(0.10–3.65)	0.583
	PRR16:High	0.68	(0.11–4.11)	0.672
	TRPM8:High	0.80	(0.13–4.81)	0.806
	KLK3:High	0.91	(0.15–5.54)	0.923
	KLK2:High	0.91	(0.15–5.54)	0.923
	GLYATL1:High	0.91	(0.15–5.54)	0.923
	EYA4:High	1.02	(0.17–6.11)	0.986
	FOLH1:High	1.02	(0.17–6.11)	0.986
	SOX2:High	1.12	(0.19–6.72)	0.904
	PPFIA2:High	1.15	(0.19–6.96)	0.880
	STEAP1:High	1.15	(0.19–6.96)	0.880
	GHR:High	1.15	(0.19–6.96)	0.880
	AR:High	1.15	(0.19–6.96)	0.880
	TMPRSS2:High	1.15	(0.19–6.96)	0.880
	EPCAM:High	1.15	(0.19–6.96)	0.880
	RORC:High	1.61	(0.27–9.78)	0.602
	WNT5A:High	2.08	(0.34–12.59)	0.425
	BRCA1:High	2.08	(0.34–12.59)	0.425
	BRCA2:High	2.08	(0.34–12.59)	0.425
	FAM107A:High	2.08	(0.34–12.59)	0.425
	TDRD1:High	2.08	(0.34–12.59)	0.425
	TLCD1:High	2.08	(0.34–12.59)	0.425
	AMACR:High	2.08	(0.34–12.59)	0.425
	SLC25A33:High	2.08	(0.34–12.59)	0.425
	NAALADL2:High	2.08	(0.34–12.59)	0.425
	SRD5A1:High	2.08	(0.34–12.59)	0.425
	FANCA:High	2.08	(0.34–12.59)	0.425
	GNMT:High	2.08	(0.34–12.59)	0.425
	PMEPA1:High	2.08	(0.34–12.59)	0.425
	TOP2A:High	2.08	(0.34–12.59)	0.425
	RRM2:High	2.08	(0.34–12.59)	0.425
	KIF11:High	2.08	(0.34–12.59)	0.425
	KIF20A:High	2.08	(0.34–12.59)	0.425
	TERT:High	2.08	(0.34–12.59)	0.425
	CCNE2:High	2.08	(0.34–12.59)	0.425
	PAX8:High	2.08	(0.34–12.59)	0.425
	BIRC5:High	2.08	(0.34–12.59)	0.425
	ADAMTS9:High	2.08	(0.34–12.59)	0.425
	ERG:High	2.08	(0.34–12.59)	0.425
	AGR2:High	2.30	(0.38–13.84)	0.364
	ARv7:High	2.45	(0.40–15.17)	0.334
	AOX1:High	2.61	(0.43–15.83)	0.296
	FAT1:High	2.61	(0.43–15.83)	0.296
	VSTM2L:High	2.61	(0.43–15.83)	0.296
	FOXA1:High	3.90	(0.62–24.72)	0.148
	TFF1:High	3.90	(0.62–24.72)	0.148
	TUSC3:High	3.90	(0.62–24.72)	0.148
	HOXB13:High	3.90	(0.62–24.72)	0.148
	GRHL2:High	3.90	(0.62–24.72)	0.148
	SIM2:High	3.90	(0.62–24.72)	0.148
	ITGBL1:High	3.90	(0.62–24.72)	0.148
	WNT7B:High	3.90	(0.62–24.72)	0.148
	PRSS8:High	7.21	(0.77–67.82)	0.084
	KRT14:High	7.21	(0.77–67.82)	0.084
	EN2:High	7.48	(0.47–119.82)	0.155
	HOXC6:High	10.97	(1.17–102.65)	0.036
	DLX1:High	10.97	(1.17–102.65)	0.036
	AKR1C3:High	10.97	(1.17–102.65)	0.036
	MYO6:High	10.97	(1.17–102.65)	0.036

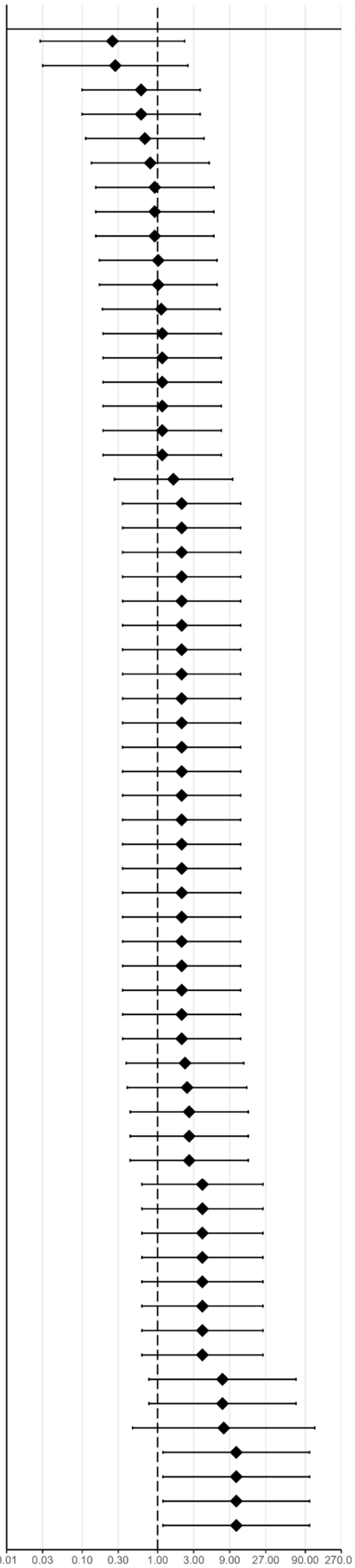

Figure S12) The prognostic value of genes in the complete gene panel on PFS in the chemotherapy cohort. A multivariate model was used to determine risk for progression (HR), 95% confidence intervals, and statistical significance (*p*). Survival statistics: HR, Hazard ratio; CI, Confidence interval; *p*, *p*-value.
