# Supplementary material for: Transcriptome Profiling of Circulating Tumor Cells to Predict Clinical Outcomes in Metastatic Castration-Resistant Prostate Cancer
Source: Int J Mol Sci. 2023 May 19;24(10):9002. doi: 10.3390/ijms24109002 (PMC10218842; doi:10.3390/ijms24109002)
Supplement: Supplementary file 1 [file ijms-24-09002-s001.zip › Supplementary Tables IJMS_20230518.pdf]

Supplementary Tables

Table S1. CTC transcriptome gene panel	
**Gene**	**Relevance to PCa**
ADAMTS9	ADAMTS9 is a potential biomarker for liquid biopsies in PCa.
AGR2	AGR2 promotes metastasis in PCa.
AKR1C3	AKR1C3 facilitates intratumoral androgen biosynthesis and androgen receptor activation.
AMACR	AMACR has been found upregulated in various PCa databases, including the PRAD TCGA database.
AOX1	AOX1 expression is correlated with biochemical recurrence.
AR	AR-signaling drivers both castration sensitive and insensitive PCa.
ARv7	AR splice variant 7 is insensitive to available ARSIs.
BIRC5	BIRC5 is upregulated in primary and metastatic PCa.
BMP6	BMP6 is overexpressed in PCa and associates with invasiveness.
BRCA1	Tumor suppressor, DNA damage response.
BRCA2	Tumor suppressor, DNA damage response.
CCNE2	CCNE2 promotes proliferation of PCa.
DLX1	DLX1 is a well-established biomarker for PCa.
EN2	EN2 is secreted in PCa but not normal prostate tissue.
EPCAM	EPCaM is an epithelial cell marker and a putative target in CTC technologies such as CellSearch.
ERG	ERG overexpression modulates transcription to drive PCa development.
EYA4	Deregulated EYA4 expression promotes progression in various cancers.
FAM107A	FAM107A expression is deregulated in PCa and is potential biomarker and therapeutic target.
FANCA	Germline FANCA alterations increase sensitivity to DNA damage repair agents.
FAT1	FAT1 regulates apoptosis in PCa.
FOLH1	FOLH1 is expressed in AR-positive, but not in AR-negative CRPC.
FOXA1	Facilitates AR-chromatin interactions and enables binding of AR to noncanonical sites.
GHR	GHR drives PCa progression.
GLYATL1	GLYATL1 is androgen regulated and upregulated in PCa.
GNMT	GNMT promotes PCa proliferation by regulating apoptosis.
GRHL2	GRHL2 is an AR coregulator with an essential role in the oncogenic AR signaling axis.
HOXB13	HOXB13 regulates AR expression as well as AR/ARv7 chromatin binding in mCRPC.
HOXC6	HOXC6 is an androgen-independent AR cofactor.
ITGBL1	ITGBL1 promotes EMT, invasion and migration in PCa.
KIF11	KIF11 is a prognostic marker for survival in mCRPC.
KIF20A	KIF20A promotes progression to mCRPC via autocrine activation of the AR.
KLK2	Canonical target of AR signaling.
KLK3	Canonical target of AR signaling.
KRT14	KRT14 expression is a marker for basal PCa cells.
KRT5	KRT5 positive and KRT14 negative PCa cells have a unique phenotype and are androgen independent.
MYO6	MYO6 promotes cellular motility and PCa cell invasiveness.
NAALADL2	NAALADL2 promotes metastatic phenotypes in PCa.
NKX3-1	Collaborates with the AR in androgen dependent PCa.
PAX8	Expression of fusion gene PAX8-GLIS3 dysregulates SHH signaling.

PCDHB2	PCDHB2 has been found upregulated in various PCa databases, including the PRAD TCGA database.
PMEPA1	PMEPA1 is potential biomarker and therapeutic target in PCa.
PPFIA2	PPFIA2 is a marker for aggressive PCa.
PRR16	PRR16 has been found upregulated in various PCa databases, including the PRAD TCGA database.
PRSS8	Is aberrantly expressed in PCa.
RORC	RORC is upregulated in mCRPC.
RRM2	RRM2 is involved with DNA damage repair and associates with poor clinical outcomes in PCa.
SIM2	SIM2 promotes the onset and progression of PCa.
SLC25A33	SLC25A33 promotes survival of malignant cells by preventing mitochondrial dysfunction.
SOX2	SOX2 promotes metastatic progression via metabolic reprogramming.
SPOCK3	SPOCK3 expression correlates with PFS in PCa.
SRD5A1	SRD5A1 facilitates intratumoral androgen biosynthesis and androgen receptor activation.
STEAP1	STEAP1 is overexpressed in PCa and the target of many emerging immunotherapies.
TDRD1	TDRD1 serves as a potential biomarker due to its overexpression in the majority of PCa cases.
TERT	High TERT expression associates with disease recurrence in PCa.
TFF1	Trefoil factors (TFFs) enhance proliferation, invasion, metastasis, and angiogenesis and inhibit apoptosis.
TLCD1	TLCD1 has been found upregulated in various PCa databases, including the PRAD TCGA database.
TMPRSS2	TMPRSS2 drives ERG overexpression in 55% of PCa cases.
TMPRSS2-ERG	Fusion of TMPRSS2 to ERG drives ERG overexpression.
TOP2A	TOPA2 promotes PCa progression via collaboration with the AR.
TRPM8	TRPM8 is a potential therapeutic target in androgen dependent PCa.
TUSC3	A tumor suppressor gene.
VSTM2L	VSTM2L expression is prognostic in various cancers and correlates with immune cell infiltration.
WNT5A	WNT5A1 induces dormancy in PCa cells.
WNT7B	WNT7B is AR regulated and promotes androgen-independent growth in CRPC.

Table S2. Baseline blood parameters of 'Group 1' and 'Group 2'

**Characteristic, mean (range)**	**Group 1**	**Group 2**
Lymphocytes (10^9/L)	1.3 (0.6-2.2)	1.5 (0.7-2.6)
Leukocytes (10^9/L)	6.7 (3-11.5)	9.2 (5-11.5)
Neutrophils (10^9/L)	4.7 (2.1-9.6)	4.4 (2.5-9.6)
Thrombocytes (10^9/L)	234.6 (116-359)	250 (119-359)
Alkaline phosphatase (U/L)	334.5 (67-1866)	103.6 (54-229)
Hb (mmol/L)	8.0 (6.6-9.4)	8.3 (6.7-9.2)
LD (U/L)	283.5 (123-663)	202.2 (111-663)
PSA μg/L	215.8 (3.6-1600)	40.8 (1.2-130)
Testosterone nmol/L	<0.4	<0.4 (<0.4-0.7)

**Table S3. Baseline blood parameters of 'ARSI 1' and 'ARSI 2'**		
**Characteristic, mean (range)**	**ARSI 1**	**ARSI 2**
Lymphocytes (10^9/L)	1.3 (0.6-2)	1.8 (0.9-3.1)
Leukocytes (10^9/L)	7 (4.9-9.5)	10.3 (4.8-68)
Neutrophils (10^9/L)	4.9 (2.7-8.1)	4 (2.5-6.1)
Thrombocytes (10^9/L)	251.7 (186-323)	257.2 (190-418)
Alkaline phosphatase (U/L)	255.3 (79-701)	99.9 (54-229)
Hb (mmol/L)	8.2 (7.3-9.4)	8.3 (6.7-9.2)
LD (U/L)	254.5 (188-317)	188.9 (111-257)
PSA µg/L	130.1 (6.7-470)	39.8 (1.2-180)
Testosterone nmol/L	<0.4	<0.4

**Table S4. Baseline alkaline phosphatase and PSA per therapy**		
**Cohort**	**Alk Phos (U/L)**	**PSA (µg/L)**
Therapy agnostic, mean (range)	195.9 (54-1866)	110.8 (1.2-1600)
ARSI, mean (range)	142.3 (54-701)	64.4 (1.2-470)
Chemotherapy, mean (range)	208.1 (54-1016)	81.5 (4.8-450)
Immuno, mean (range)	120 (103-137)	82 (44-120)
Radioligand	110.3 (82-178)	81 (39-160)
PARPi, mean (range)	99 (94-104)	120 (110-130)
Active surveillance	715.7 (63-1866)	557.3 (24-1600)

**Table S5. Healthy volunteer demographics**	
**Characteristics**	**Statistics**
Num. of samples	29
Age, median (range)	50 (22-71)
Sex, Num. (%)	
Male	19 (66%)
Female	10 (34%)
Men aged > 50, Num. (%)	9 (47%)
Men aged < 50, Num. (%)	10 (53%)

Table S6. TSO500 PCa gene panel
AKT1
AKT2
AKT3
AR
ATM
ATR
BARD1
BRAF
BRCA1
BRCA2
BRIP1
CCND1
CDK12
CDK4
CDK6
CDKN2A
CHEK1
CHEK2
ERCC2
FANCL
MAP2K1
MAP2K2
MAP2K4
MAP3K1
MLH1
MSH2
MSH6
NBN
PALB2
PIK3CA
PIK3CB
PIK3R1
PMS2
POLE
PPP2R2A
PTEN
RAD51B
RAD51C
RAD51D
RAD54L
RAF1
RB1
SPOP
TP53
